# Chromosome Scaffold is a Double-Stranded Assembly of Scaffold Proteins

**DOI:** 10.1038/srep11916

**Published:** 2015-07-01

**Authors:** Rawin Poonperm, Hideaki Takata, Tohru Hamano, Atsushi Matsuda, Susumu Uchiyama, Yasushi Hiraoka, Kiichi Fukui

**Affiliations:** 1 Department of Biotechnology, Graduate School of Engineering, Osaka University, 2-1 Yamadaoka, Suita, Osaka 565-0871, JAPAN; 2 Frontier Research Base for Global Young Researchers, Graduate School of Engineering, Osaka University, 2-1 Yamadaoka, Suita, Osaka 565-0871, JAPAN; 3 Graduate School of Frontier Biosciences, Osaka University, 1-3 Yamadaoka, Suita, Osaka 565-0871, JAPAN; 4 Advanced ICT Research Institute Kobe, National Institute of Information and Communications Technology, 588-2 Iwaoka, Iwaoka-cho, Nishi-ku, Kobe 651-2492, JAPAN

## Abstract

Chromosome higher order structure has been an enigma for over a century. The most important structural finding has been the presence of a chromosome scaffold composed of non-histone proteins; so-called scaffold proteins. However, the organization and function of the scaffold are still controversial. Here, we use three dimensional-structured illumination microscopy (3D-SIM) and focused ion beam/scanning electron microscopy (FIB/SEM) to reveal the axial distributions of scaffold proteins in metaphase chromosomes comprising two strands. We also find that scaffold protein can adaptably recover its original localization after chromosome reversion in the presence of cations. This reversion to the original morphology underscores the role of the scaffold for intrinsic structural integrity of chromosomes. We therefore propose a new structural model of the chromosome scaffold that includes twisted double strands, consistent with the physical properties of chromosomal bending flexibility and rigidity. Our model provides new insights into chromosome higher order structure.

Chromosome condensation is crucial to ensure the fidelity of chromosome segregation during cell division. However, how chromosomes are organized in mitosis still remains one of the most important enigmas in cell biology. In the late 1970s, Laemmli and colleagues observed a backbone structure in chromosomes after depletion of histone proteins. The backbone was positioned along the chromosome axes, and thus termed the ‘chromosome scaffold’^1^. The axially-positioned chromosome scaffold of both chromatids mainly comprises non-histone proteins: so-called scaffold proteins, including condensin, topoisomerase IIα (Topo IIα), and kinesin family member 4 (KIF4)^2^,^3^,^4^,^5^. Various approaches have been tried to elucidate chromosome scaffold structure. Initial electron microscopy (EM) observations of histone-depleted chromosomes suggested that the chromosome scaffold is a network structure^1^. Immunofluorescence has permitted identification of condensin and Topo IIα within the axial regions of mitotic chromosomes, with their alternating distribution patterns leading to a ‘barber pole’ model^6^. However, due to the diffraction limit of light, the detailed distribution patterns of each scaffold protein are still ambiguous. Broad axial localization of condensin and Topo IIα have also been observed using EM with immunogold labelling^7^,^8^. In contrast, micromechanical force measurements suggest that chromosomes have no rigid scaffold^9^. Due to these discrepancies, the structure of the chromosome scaffold still remains elusive.

The structural and functional roles of the main scaffold proteins, condensin, Topo IIα, and KIF4, have been widely studied^2^,^3^,^4^,^5^,^6^,^10^,^11^,^12^,^13^, but it is unclear how these proteins organize the scaffold and regulate chromosome condensation. Here, we used 3D-SIM, which has a resolution (~120 nm lateral) that is twice the optical diffraction limit^14^, to visualize the localization of each scaffold protein and the structure of the scaffold in metaphase chromosomes. FIB/SEM was used in conjunction because this enables direct observation of protein localization inside a chromosome at scanning EM resolution^15^. Using these new methods, we revealed that the chromosome scaffold exhibited a scaffold protein distribution comprising two main lateral strands within the chromatid axis. We also examined the structural role of scaffold proteins by RNAi and found that proper assembly of the scaffold proteins is important for the double-stranded scaffold organization as well as chromosome morphology. Furthermore, we found that the chromosome scaffold could be altered and restored in parallel with changes of chromosome morphology, suggesting the structural role of scaffold proteins contributes to the intrinsic integrity of chromosomes. Taken together, our present studies pave the way for understanding chromosome higher order structure in relation to the structural and functional role of the chromosome scaffold.

## Results

### Double-stranded chromosome scaffold (DCS) revealed by super-resolution microscopy, 3D-SIM

**Figure 1:**
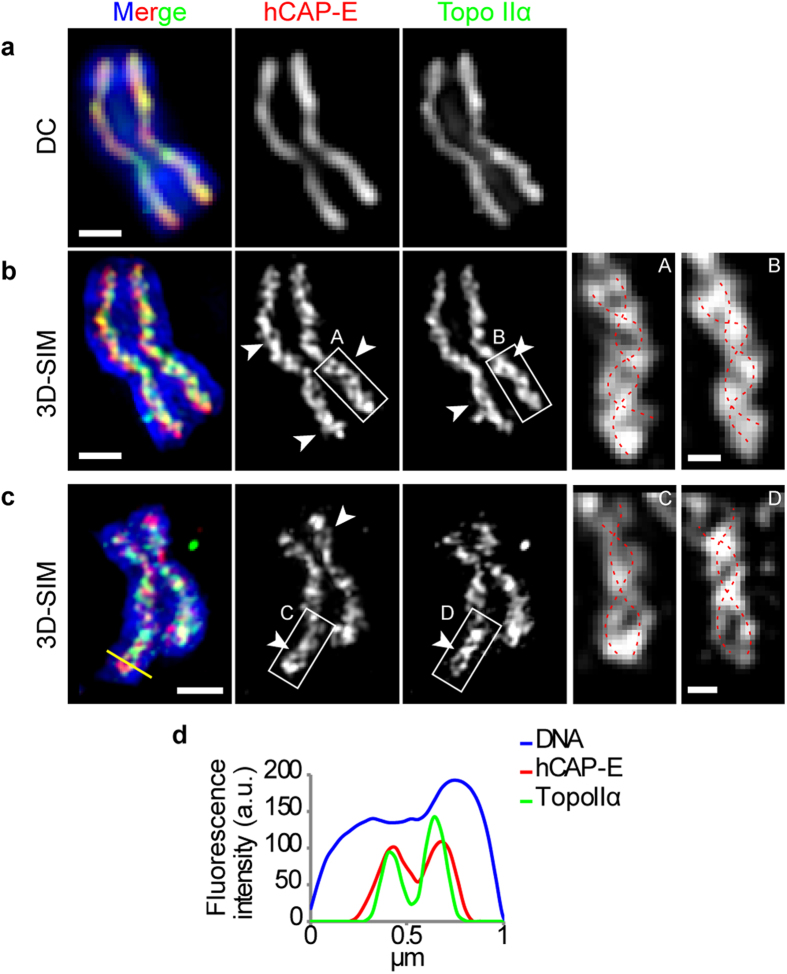
Localization of hCAP-E and Topo IIα revealed by 3D-SIM. **a**–**c**, Maximum intensity projections of z-stack images. Scale bars, 1 μm. **a**, wide-field microscopy applying deconvolution imaging of PA chromosome immunostained for hCAP-E and Topo IIα. **b**, 3D-SIM image of the same PA chromosome as **a**. **c**, 3D-SIM image of HeLa-wt metaphase chromosome immunostained for hCAP-E and Topo IIα. Arrowheads indicate the double strands. Insets show magnified views of the white boxes in **a** and **b** as indicated. Scale bars, 250 nm. Red dotted lines represent double strands of chromosome scaffold. DNA is shown in blue. **d**, RGB line profile of yellow path in **c**.

To investigate the localization of chromosomal scaffold proteins, an initial immunofluorescence study of over 100 polyamine-isolated human chromosomes (PA chromosomes) was performed using wide-field microscopy with deconvolution. We found that hCAP-E (a condensin subunit), Topo IIα, and KIF4 are characteristically localized at the central axis of each chromatid (Fig. 1a, Supplementary Fig. 1a) and exhibited continuously alternating distributions, consistent with previous reports^4^,^5^,^6^. However, when these chromosomes were imaged by 3D-SIM, we found that the chromosome scaffold was composed of networks of hCAP-E, Topo IIα and KIF4 along the chromatid axes (Fig. 1b, Supplementary Fig. 1b), reminiscent of the fibrous network found by transmission EM^1^. The same result was observed with HeLa-wild type (HeLa-wt) spread chromosomes (Fig. 1c, Supplementary Fig. 1c). Double strands were also observed more than 80% of chromosomes examined and often appeared to be at least partially twisted around each other (Fig. 1b,c, Supplementary Fig. 1b, c). Line profiles were analyzed along the chromatid arms to confirm the bimodal distribution of scaffold proteins (Supplementary Fig. 2). We found that hCAP-E, Topo IIα and KIF4 evidently show two peaks within a single chromatid (Fig. 1d, Supplementary Fig. 1d and 2). These facts indicate the double-stranded nature of chromosome scaffold. At the same time, we also found single-peak of the intensities in more or less alternating to double-peak patterns, suggesting the twisted form of chromosome scaffold. Based on these observations by 3D-SIM, we believe that chromosome scaffold may be consisted of twisted double strands.

### 3D reconstruction of chromosome scaffold in metaphase chromosomes by FIB/SEM

**Figure 2:**
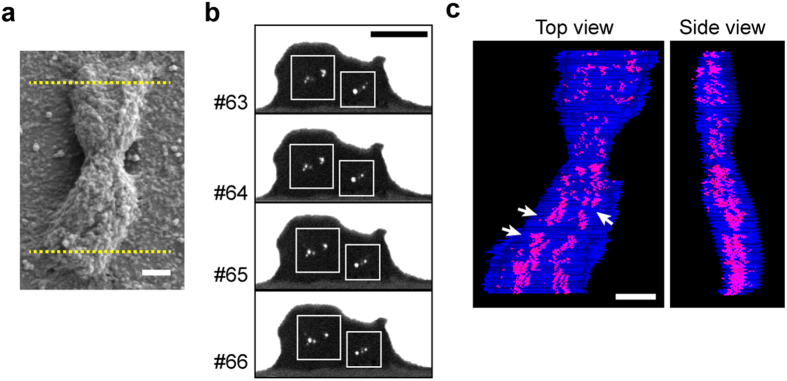
Three-dimensional reconstruction of Topo IIα localization in a PA chromosome observed by FIB/SEM. **a**, SEM image of a representative PA chromosome immunogold-labelled for Topo IIα. Lower and upper dashed lines represent starting and ending dissection points, respectively. **b**, Sequential backscatter electron images of FIB crossed-sections of PA chromosome in **a**. # represents No. of the cross-section. White boxes show two clusters of silver-enhanced nanogold particles. **c**, A 3D-model reconstructed from total cross-section series of PA chromosome in **a** by FIB/SEM. The chromosome outline and Topo IIα localization are shown as blue and red, respectively. Arrows indicate the double strands. Scale bars, 500 nm. Slices thickness were 10 nm.

To confirm the double-stranded nature of the chromosome scaffold, the distribution of scaffold proteins was observed by FIB/SEM. PA chromosomes were stained for individual Topo IIα or hCAP-E with a fluoronanogold particle-labelled antibody, followed by silver enhancement^15^. Two of Topo IIα stained chromosomes and three of hCAP-E stained chromosomes were sequentially dissected by FIB and the cross-sections were directly observed by SEM. In backscatter electron mode, in each of five chromosomes, we clearly observed two major clusters of silver-enhanced fluoronanogold signals within the chromatid, representing Topo IIα (Fig. 2b, white boxes) and condensin localization (Supplementary Fig. 3). Some sections showed diffusion of scaffold proteins in a chromatid, although signals were mainly distributed at the center of the chromatid. These results suggest the existence of DCS in the chromosomes.

To further examine the distribution of Topo IIα along the chromosome axes, after sequential dissection and observation of a Topo IIα-stained chromosome by FIB/SEM, complete sequential images of the representative chromosome (Fig. 2a) were reconstructed into a three-dimensional (3D) model (Fig. 2c). This confirmed the axial localization of Topo IIα and the presence of a DCS (Fig. 2c, arrows); the double strands in the scaffold were also seemed to be twisted (Fig. 2c). These results confirm the axial localization of scaffold protein in the chromosome and the nature of twisted double-stranded chromosome scaffold.

### Structural contributions of condensin, Topo IIα and KIF4 to the DCS

**Figure 3:**
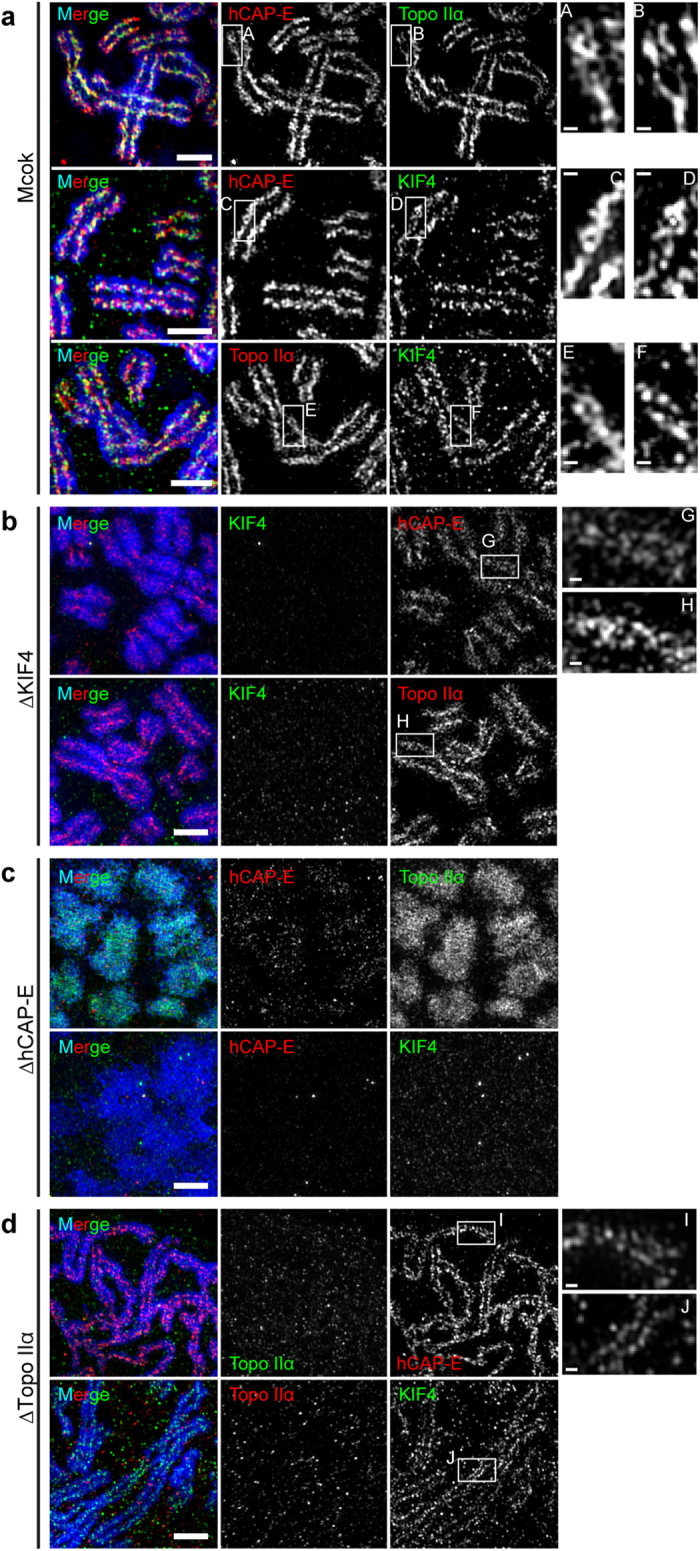
Depletion of hCAP-E or Topo IIα, but not KIF4, disrupts construction of a double-stranded chromosome scaffold (DCS) and proper chromosome organization. **a**,**b**,**c**, Maximum intensity projections of z-stack images obtained by 3D-SIM of KIF4-, hCAP-E- and Topo IIα-depleted HeLa-wt cells, respectively, immunostained for targeted scaffold proteins as indicated. Scale bars, 2 μm. DNA is shown in blue. Insets show magnified views of the white boxes in **a**,**b** and **d** as indicated. Scale bars, 250 nm.

To investigate which scaffold proteins are required for construction of the DCS, individual scaffold proteins were depleted from HeLa-wt cells using siRNA (Supplementary Fig. 4). DCS was often visualized by 3D-SIM observations in mock-treated chromosomes (Fig. 3a), similar to the results in non-transfected HeLa-wt mitotic cells (Fig. 1c, Supplementary Fig. 1c). After KIF4 depletion, hCAP-E and Topo IIα had slightly more dispersed distributions in broader chromosomes (Fig. 3b) compared to mock-treated chromosomes (Fig. 3a); the DCS was still observed (Fig. 3b, insets). This indicates that KIF4 may not be directly involved in formation of the DCS. In hCAP-E-depleted cells, chromosomes had a highly distorted structure and the chromosome scaffold was not constructed (Fig. 3c). Topo IIα was highly diffused throughout the chromosomes, while KIF4 was greatly reduced with depletion of hCAP-E (Fig. 3c, Supplementary Fig. 4b). Because KIF4 knockdown also reduced hCAP-E level on the chromosomes (Fig. 3b, Supplementary Fig. 4b). These results indicate the interdependency of KIF4 and condensin for localization to the chromosome scaffold^5^. In Topo IIα-depleted cells, chromosomes had an elongated shape. Although, a single dotted line liked distribution of hCAP-E and KIF4 was observed on the chromatid axes, no double strands were detected (Fig. 3d). These results signify that condensin is primarily important for construction of the chromosome scaffold^10^, and that Topo IIα should be responsible for construction the DCS, because, without Topo IIα, although the chromosome scaffold could be established, double strands could not be observed (Fig. 3d).

Based on these results, both condensin and Topo IIα contribute more essentially to DCS organization and maintenance of chromosome morphology than KIF4. Although KIF4 is not necessary for DCS formation, it is still required for chromosome condensation^4^,^5^ because chromosomes were wider in the KIF4 depleted cells (Supplementary Fig. 5). Due to interdependency of KIF4 and condensin, therefore, KIF functions may just support recruitment of condensin to the chromosomes scaffold and supplement condensin function of lateral compaction^5^. Furthermore, loss of any scaffold protein basically resulted in defective localization of the remaining scaffold proteins and led to abnormal chromosome morphology (Fig. 3b–d). This suggests that appropriate assembly of scaffold proteins, possibly via protein-protein interaction^4^,^16^,^17^, is important for the formation of a DCS and proper chromosome morphology.

### Adaptability of scaffold proteins in relation to the chromosome condensation

**Figure 4:**
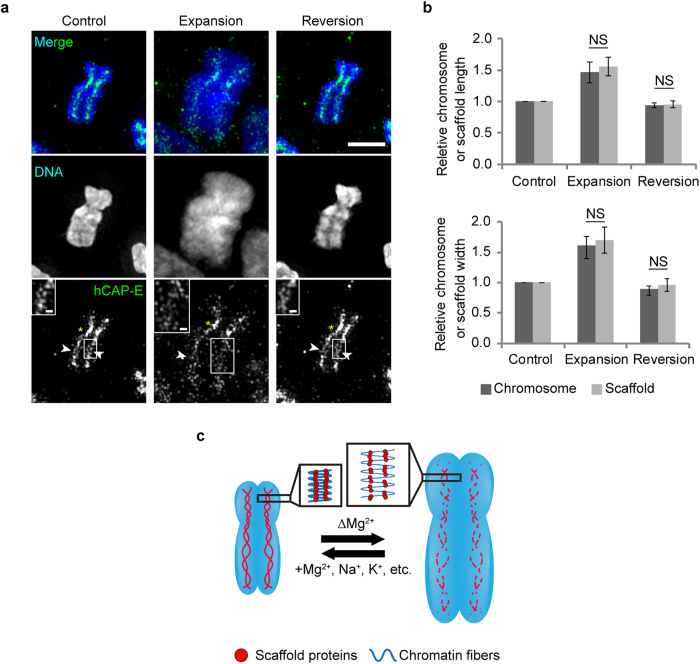
The double-stranded chromosome scaffold (DCS) is highly adaptable in relation to chromosome changes. **a**, Maximum intensity projections of z-stack images obtained by 3D-SIM of unfixed PA chromosome treated with PBS (Control), 1 mM EDTA (Expansion), or H-Mg (Reversion). Scale bar, 2 μm. Arrowheads indicate double strands. Asterisks indicate the centromeric region. Insets show magnified views of white boxes. Scale bar, 250 nm. **b**, Relative chromosome or chromosome scaffold length and width in each treatment. Bar denotes the mean; NS, not significant; error bar denotes standard deviation (n = 46). **c**, Schematic chromosome scaffold adaptations in relation to scaffold protein interactions and chromatin network reinforcement.

To examine the adaptability of the DCS in chromosome condensation and decondensation, we tracked the distribution of hCAP-E in unfixed PA chromosomes after removal and subsequent re-addition of Mg^2+^ for induction of chromosome decondensation and recondensation, respectively^18^,^19^. Initially, unfixed PA chromosomes maintained in PBS exhibited an authentic morphology and the hCAP-E distribution was visualized as double-stranded (Fig. 4a, Control), similar to that in fixed chromosomes (Fig. 1b,c). After substitution of PBS with 1 mM EDTA, chromosomes were swollen due to lack of Mg^2+^ (Fig. 4a, Expansion). At this stage, hCAP-E signals were still axially located in the chromatids, but the distribution pattern broadened and lengthened in parallel with expansion of chromosome width and length (Fig. 4a, Expansion). After exchange of 1 mM EDTA with HEPES-buffer containing 5 mM Mg^2+^ (H-Mg), authentic chromosome morphology was recovered and hCAP-E localization was restored (Fig. 4a, Reversion). Interestingly, at the centromeric positions, no distribution change of hCAP-E was observed, and hCAP-E fluorescence signals remained strong (Fig. 4a, bottom panel) in all conditions. These results show that chromosome scaffold organization is adaptable to different chromosome states: a broken, discontinuous organization in the expanded state versus a twisted double-strand in the compacted state (Fig. 4a). Chromosomes are, therefore, able to change its morphology reversibly and adaptably to their physiological conditions. This is consistent with micromechanical measurement results showing that chromosomes are flexible objects^9^,^20^.

Chromosome and chromosome scaffold lengths and widths in all conditions were measured and relative chromosome and chromosome scaffold lengths and widths were evaluated (Supplementary Fig. 6). The expansion and reversion ratios of chromosome and chromosome scaffold were very similar in both axial and lateral directions (Fig. 4b). When conditions were reverted, the chromosome and chromosome scaffold dimensions were similarly reverted (Fig. 4b). The capability of chromosome scaffold to restore its structure after recovery of Mg^2+^ suggests the scaffold proteins play an important role in the intrinsic structural integrity of the chromosomes.

Furthermore, K^+^ and Na^+^ also restored the authentic chromosome morphology and scaffold structure (Supplementary Fig. 7). This indicates that the restoring function of divalent cations could be substituted by univalent cations. Moreover, localizations of scaffold protein were also efficiently recovered to the authentic positions with all the reversible buffers employed in the present study (Fig. 4a, Supplementary Fig. 7). This suggests that the distribution of scaffold proteins is independent of the kind of cations, and that simple electrostatic conditions would play an essential role in the chromosome condensation and decondensation.

## Discussion

### Double-stranded chromosome scaffold (DCS) and its structural function

Chromosome scaffolds and their scaffold proteins have been studied for more than 40 years. However, the precise distribution and function of the scaffold proteins have been controversial due to the dynamic nature of the chromosome and the limitations of analytical methods. By employing a high-resolution structural analysis using 3D-SIM and FIB/SEM, the axial distributions of scaffold proteins in metaphase chromosomes comprising DCS has now been revealed. The double-stranded scaffold, which is reminiscent of the fibrous network previously found by EM observation^1^, is inconsistent with the contemporary consensus of a continuous single axis with alternating distributions as previously inferred from ordinary optical microscopy^5^,^6^. Although, a chromosome scaffold fibrous network has been detected previously by EM observations^1^,^21^, the harsh treatment during sample preparation precluded certainty about the native structure of the chromosome scaffold. Therefore, we used a simple fixation method with freshly prepared formaldehyde to maintain the chromosome structure as intact as possible. As a result, we have revealed the DCS within chromosomes for the first time.

Understanding why the DCS exists is important for understanding chromosome structure. It has been suggested that decreasing the chromosome scaffold axial radius may enhance chromosome flexibility^20^. As a result, chromosomes with thin axial scaffolds are bent more easily than those with a single thick axis. The presence of multiple thin axes may be well-suited to support other properties relating to chromosome strength. Thus, the function of the DCS may be to simultaneously satisfy chromosome bending elasticity and stiffness by having the double strands twist around each other, as these physical properties are essential for chromosome movement during cell division^20^.

### The role of scaffold proteins and the formation of chromosome scaffold

We found that the configuration of the chromosome scaffold was well preserved after the chromosome structural transitions of expansion and reversion (Fig. 4a, Supplementary Fig. 7). This strongly supports previous findings on the role of scaffold proteins in the intrinsic structural integrity of chromosomes^5^,^10^. Furthermore, this suggests that the chromosome scaffold has an anchoring function, in order to maintain the authentic structure of chromosome formed by the appropriate assembly of scaffold proteins. This is evidenced by scaffold protein depletion causing defective chromosome scaffold formation (Fig. 3). Moreover, chromosomes lacking condensin or KIF4 are not able to recover their X-shape after chromatin unfolding^5^,^10^.

During chromosome expansion and reversion in the absence or presence of cations, relative chromosome and chromosome scaffold length and width ratios remained essentially constant (Fig. 4b), while positions of hCAP-E were linked with the expansion and reversion of DNA and thus the chromosome (Fig. 4a, Supplementary Fig. 7). This suggests a strong association of scaffold proteins with specific DNA regions, presumably SARs/MARs^22^ found along chromatid axes and colocalized with Topo IIα^23^. Since repetitive DNAs can self-assemble in the presence of Mg^2+^ 24, they might form an axial compacted chromatin network separated from the chromatin loops^25^. Thus, DNA itself may also act to preserve the chromosome scaffold configuration by its own self-interaction, which is further support for the accumulation of and interaction between the scaffold proteins. This is confirmed by the consistent enrichment of hCAP-E in the centromeric region that contains large amount of repetitive DNAs, despite the absence of Mg^2+^ (Fig. 4a, Expansion), suggesting maintenance of scaffold protein accumulation by highly compacted chromatin fibers. Thus, we propose the formation of twisted double strands of chromosome scaffold requires assembly of scaffold proteins via protein-protein interactions, and that this is promoted by compaction of chromatin fibers^26^ that include repetitive DNAs in the presence of cations (Fig. 4c).

### Chromosome formation by cooperation of scaffold proteins and cations

Our results are consistent with a two-stage folding model that derived from chromosome conformation capture data^27^. In the linear compaction process, cations neutralize DNA negative charges to regulate chromosome compaction, and to induce self-organize of DNA^24^ to promote the interaction of the DNA-bound scaffold proteins. The chromosome scaffold is then primarily established and acts to determine and maintain the authentic chromosome shape. In the axial compression process, scaffold proteins are responsible for the large-scale organized compaction of chromatin fibers via their enzymatic activities to form a highly compacted chromosome. Therefore, without establishment of the chromosome scaffold, chromatin might still be condensed^5^, presumably by cations, but would form abnormal chromosomes.

In conclusion, the DCS has a regular structure, but the structure is highly adaptable because scaffold proteins are not covalently bound to each other and follow the movement of DNA. Therefore, the structure of the chromosome scaffold can be transformed under different conditions (data not shown). Accordingly, the assembly of scaffold proteins, especially condensin and Topo IIα, facilitated by DNA compaction in the presence of cations, is necessary to establish the DCS and maintain chromosome structure. These results support previous findings on the role of scaffold proteins in the intrinsic structural integrity of chromosomes^5^,^10^. Thus, our findings provide a basis for understanding chromosome integrity and explain the dynamic aspects of chromosome properties.

## Materials and Methods

### Cells culture and synchronization

HeLa-wt cells were cultured in complete medium (Dulbecco’s modified Eagle’s medium (DMEM; GIBCO BRL) supplemented with 10% fetal-bovine serum (FBS; Equitech-Bio)) at 37 °C under 5% CO_2_. At 10–15% confluence, HeLa cells were synchronized in complete medium containing a final concentration of 2.5 mM thymidine (Sigma). After thymidine blocking for 24 h, thymidine was removed by washing twice with PBS (137 mM NaCl, 2.7 mM KCl, 10 mM Na_2_HPO_4_, 2 mM KH_2_PO_4_ pH 7.4) and the cells were then recultured in fresh complete medium for 12 h to release cells. HeLa cells were synchronized in complete medium containing thymidine (final concentration 2.5 mM) for 18 h. After the second thymidine blocking, thymidine was removed by washing twice with PBS and the cells were treated with fresh complete medium to release cells. To obtain metaphase cells, after 8 h reculturing, 0.1 μg/ml colcemid was added and the cells were incubated for 3 h before harvesting.

### Antibodies

The following primary antibodies were used for indirect immunostaining and immunoblotting: mouse monoclonal anti-Topo IIα (1:50; Topogen); mouse monoclonal anti-Topo IIα (1:1,000; Abnova); rabbit polyclonal anti-hCAP-E (1:100); goat polyclonal anti-KIF4A (1:50; Thermoscience); and mouse monoclonal anti-histone H3 (1:2,000; Millipore).

### Immunostaining and 3D-SIM observation

HeLa-wt cells treated with 75 mM KCl for 15 min at 37 °C or PA chromosomes prepared from HeLa S3^28^ were spun onto coverslips (Zeiss) coated with poly-L-lysine (Sigma) by cytocentrifugation (Shandon Cytospin 4, Thermo) at 1,300 rpm for 10 min. Immunostaining was performed as described^6^ with some modifications. Cells were fixed with 2% formaldehyde (freshly prepared from *para*-formaldehyde) in PBS for 15 min and permeabilized using 0.2% Triton X-100 in PBS for 10 min. Cells were blocked with 3% BSA in PBS for 1 h. Primary and secondary antibody reactions were then performed under the same conditions at room temperature for 1 h. DNA was counterstained with Hoechst 33342. Samples were mounted in Vectorshield mounting medium (Vector Laboratories). Images were taken with DeltaVision OMX ver. 3 (GE Healthcare) using a 1.4 NA PlanApo 100× oil-immersion objective (Olympus), immersion oil (refractive index 1.518); and a Cascade II:512 camera (Photometrics). The z-section distance was 125 nm and the total z-section thickness was set at 4–6 μm. To reconstruct high-resolution images, raw images were computationally processed by softWoRx 6.0 Beta 19, using custom optical transfer functions for each wavelength with Wiener filter constants from 0.002 to 0.012. Channel alignment was used to correct for chromatic shift. The brightness and contrast of images were adjusted using ImageJ (National Institutes of Health, USA).

### Immunogold labelling and sample preparation for FIB/SEM

Immunogold labelling was performed as described^15^ with some modifications. PA chromosomes were dropped onto an aluminum foil substrates and treated with 0.5% triton X-100 in XBE2 (10 mM HEPES pH 7.7, 100 mM KCl, 5 mM EGTA, 2 mM MgCl_2_) for 10 min. Chromosomes were then blocked in 1% BSA in XBE2 for 30 min and incubated with Topo IIα antibody (1:50; Topogen) or hCAP-E (1:100) for 1 h. After washing, chromosomes were incubated with anti-mouse FluoroNanogold®or anti-rabbit FluoroNanogold® (1:200; Nanoprobes) for 1 h. The chromosomes were post-fixed with 2.5% glutaraldehyde in XBE5 (10 mM HEPES pH 7.7, 100 mM KCl, 5 mM EGTA, 5 mM MgCl_2_). After washing, samples were silver-enhanced (HQ silver, Nanoprobes). The immunogold-labelled chromosomes were then treated with 2% osmium tetroxide and extensively washed with ultrapure water. Sample dehydration was carried out using an ethanol series (70%, 100% and 100%) and then treatment with 3-methylbutyl acetate and critical point drying, after which osmium coating was performed.

### FIB/SEM observation

FIB was performed using an Auriga 60 FIB/SEM system (Zeiss). The FIB consists of Ga^+^ ions accelerated by a voltage of 30 kV. The SEM column was mounted on the top of the system chamber and the FIB column was at an angle of 54°. The FIB/SEM system was equipped with a gas injection system loaded with platinum and carbon gas precursors. Platinum and carbon coating were performed before FIB/SEM experiments. In the cut-and-view mode, sectioning was performed by FIB and images were recorded by SEM with an electron voltage of 2 kV in secondary electron and backscattering electron modes at a working distance of 5.0 mm. Specimens were tilted to an angle of 54°. Images were tilt-corrected for an undistorted surface view. To reconstruct the 3D-model, sequential images were computationally processed by IMOD 4.5.

### Analysis of chromosome scaffold adaptability: expansion and reversion

Unfixed PA chromosomes were spun onto a glass-bottom dish and blocked with 3% BSA in PBS for 1 h. Primary and secondary antibody reactions were performed under the same conditions, except for using anti-hCAP-E and Alexa 488 anti-rabbit IgG, respectively. Chromosomes were maintained in PBS in the initial step. For expansion, PBS was removed and 1 mM EDTA was added to the chromosomes. For reversion, 1 mM EDTA was removed and a reversible buffer (see below) was added. Observations were performed using 3D-SIM, as described above. The reversible buffers were H-Mg (10 mM HEPES, 5 mM MgCl_2_, pH 7.4); H-K (10 mM HEPES, 120 mM KCl, pH 7.4); and H-Na (10 mM HEPES, 20 mM NaCl, pH 7.4).

### RNA interference transfection and western blotting

The small interfering RNA (siRNA) sequences were as follows:

hCAP-E, 5′-UGCUAUCACUGGCUUAAAUTT-3′;

KIF4, 5′-GCAAUUGAUUACCCAGUUATT-3′; and

Topo IIα, 5′-AAGACUGUCUGUUGAAAGATT-3′.

For transfection, HeLa cells were incubated with 120–200 nM duplex siRNA using Lipofectamine®2000 (Invitrogen). Transfection was performed concomitantly with synchronization using thymidine and colcemid. After 72 h of incubation, cells were collected and used for analysis. Western blotting was performed as described following. Extracted proteins from cell lysates and chromatin fractions were separated by SDS-PAGE and transferred to a membrane using iBlot (Invitrogen). Blocking was performed in 2% BSA in TBST (20 mM Tris, pH 7.5, 150 mM NaCl, 0.1% (v/v) Tween 20). Primary and secondary antibodies were diluted in the same blocking solution. Immunoreactive bands of proteins were detected using 1% NBT/BCIP stock solution (Roche) in alkaline phosphatase buffer (100 mM Tris pH 9.5, 100 mM NaCl, 5 mM MgCl_2_).

## Additional Information

**How to cite this article**: Poonperm, R. *et al*. Chromosome Scaffold is a Double-Stranded Assembly of Scaffold Proteins. *Sci. Rep*. **5**, 11916; doi: 10.1038/srep11916 (2015).

## Supplementary Material

Supplementary Information
