# Supplementary material for: Chromosome Scaffold is a Double-Stranded Assembly of Scaffold Proteins
Source: Sci Rep. 2015 Jul 1;5:11916. doi: 10.1038/srep11916 (PMC4487240; doi:10.1038/srep11916)
Supplement: Supplementary Information [file srep11916-s1.pdf]

Supplementary Information

CHROMOSOME SCAFFOLD IS A DOUBLE-STRANDED ASSEMBLY OF SCAFFOLD PROTEINS

Rawin Poonperm[1], Hideaki Takata[1,2], Tohru Hamano[1], Atsushi Matsuda[3,4], Susumu Uchiyama[1], Yasushi Hiraoka[3,4] and Kiichi Fukui[1]*

[1] Department of Biotechnology, Graduate School of Engineering, Osaka University, 2-1 Yamadaoka, Suita, Osaka 565-0871, JAPAN

[2] Frontier Research Base for Global Young Researchers, Graduate School of Engineering, Osaka university, 2-1 Yamadaoka, Suita, Osaka 565-0871, JAPAN

[3] Graduate School of Frontier Biosciences, Osaka University, 1-3 Yamadaoka, Suita, Osaka 565-0871, JAPAN

[4] Advanced ICT Research Institute Kobe, National Institute of Information and Communications Technology, 588-2 Iwaoka, Iwaoka-cho, Nishi-ku, Kobe 651-2492, JAPAN

*Corresponding author: Kiichi Fukui, Contact. Tel: +81-6-6879-7440;

Fax: +81-6-6879-7441; Email: kfukui@bio.eng.osaka-u.ac.jp

Supplementary Information Guide

This file includes

Supplementary Figures and Legends S1-S7

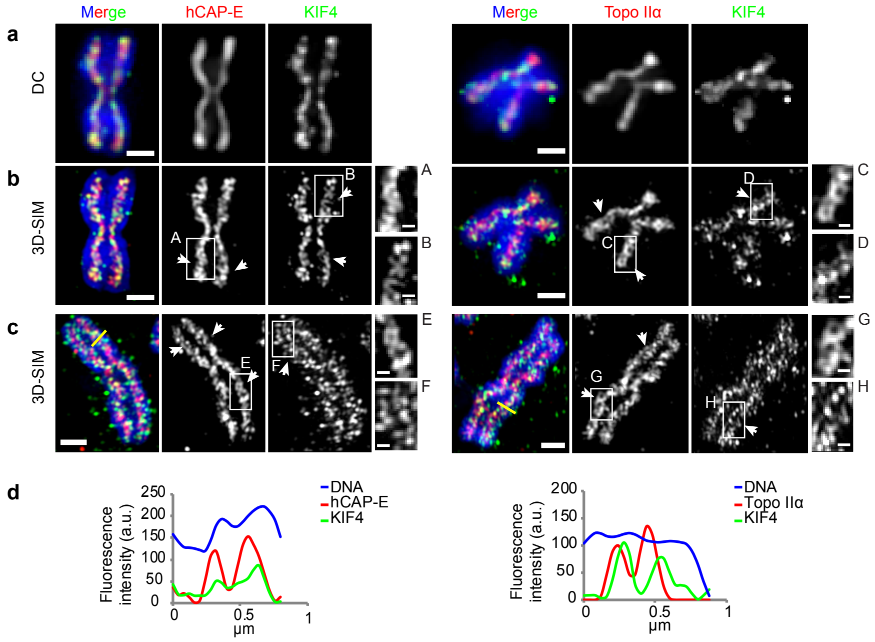

Supplementary Figure 1. All scaffold proteins have a double-stranded distribution at the chromatid axis. a-c, Maximum intensity projections of z-stack images. Scale bars, 1 µm. **a**, wide-field microscopy applying deconvolution imaging of PA chromosome immunostained for hCAP-E, Topo IIα, and KIF4 as indicated. **b**, 3D-SIM image of the same PA chromosome as **a**. **c**, 3D-SIM image of HeLa-wt metaphase chromosome immunostained for hCAP-E, Topo IIα, and KIF4 as indicated. Arrowheads indicate the double strands. KIF4 distribution is sometimes dispersed apart from the chromosome scaffold. Magnified views of white boxes in **b** and **c** are shown in insets as indicated. Scale bars, 250 nm. DNA was shown in blue. **d**, RGB line profiles of yellow paths in **c**. Detailed line profiles of **c** were shown in Supplementary Fig. 2.

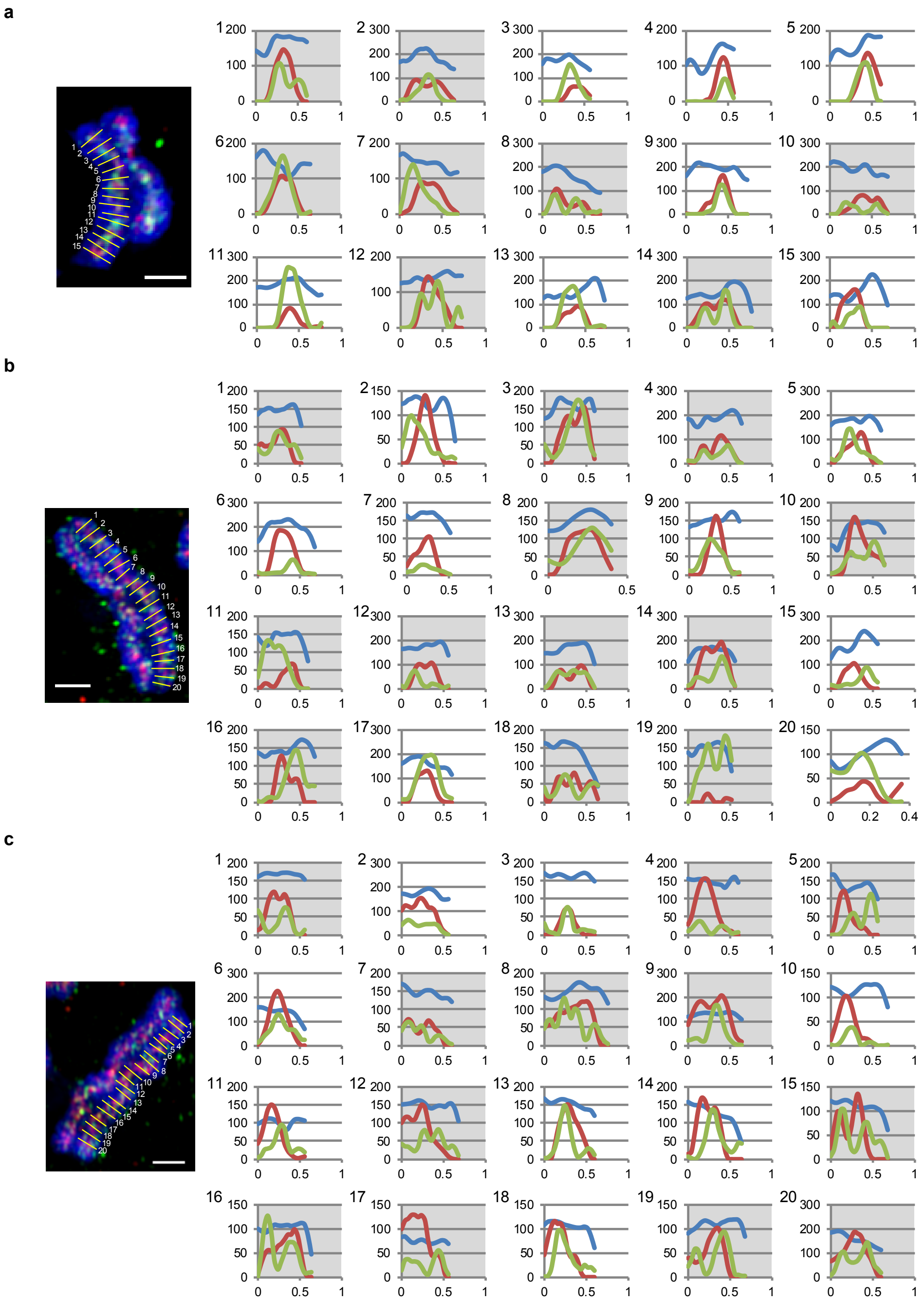

Supplementary Figure 2. Line profile plots. a, HeLa-wt metaphase chromosomes immunostained for hCAP-E (red) and Topo IIα (green). **b**, HeLa-wt metaphase chromosomes immunostained for hCAP-E (red) and KIF4 (green). **c**, HeLa-wt metaphase chromosomes immunostained for Topo IIα (red) and KIF4 (green). Scale bar, 1 μm. These are the same chromosomes from Fig. 1c and Supplementary Fig. 1c. Yellow lines with numbers indicate locations of line profile plots on the right. Line

profile plots showing double peaks of protein distributions within one chromatid were shown with gray background.

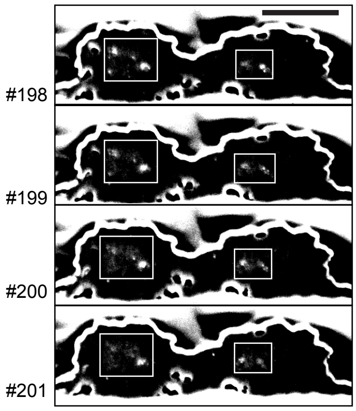

Supplementary Figure 3. Condensin localization in a PA chromosome observed by FIB/SEM. PA chromosomes stained for hCAP-E with nanogold particle-labelled antibody was observed by FIB/SEM. Sequential backscatter electron images of continuous FIB crossed-sections of PA chromosome showing hCAP-E localization as bright spots. Scale bar, 500 nm. White boxes show two clusters of silver-enhanced nanogold particles in a chromatid suggesting the double-stranded nature of the chromosome scaffold. # represents No. of the cross-section. Slice thickness was 5 nm.

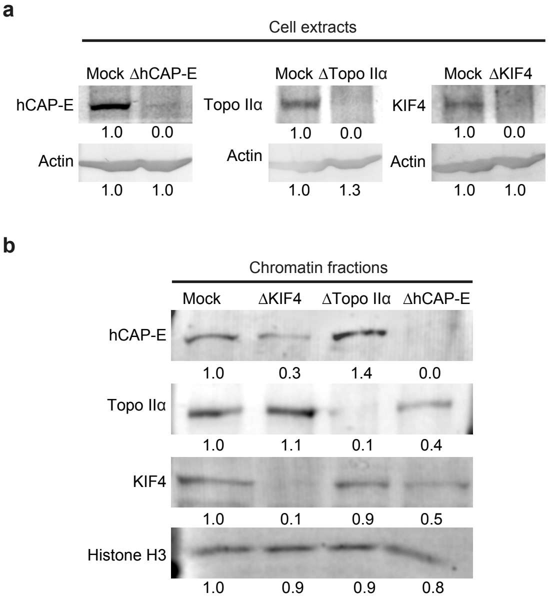

Supplementary Figure 4. Depletion of individual scaffold proteins affects other scaffold proteins. **a**, Western blot of scaffold proteins in total extracts of HeLa-wt cells confirmed the knockdown of the endogenous scaffold proteins after depletion of KIF4, Topo IIα, and hCAP-E by siRNA transfection for 72 hr. Actin was used as control. **b**, Western blot of scaffold proteins in chromatin fraction from the cells in **a**. Histone-H3 was used as control. The relative ratios of the band intensity of each lane versus mock, taking the mock as 1.0, are shown below.

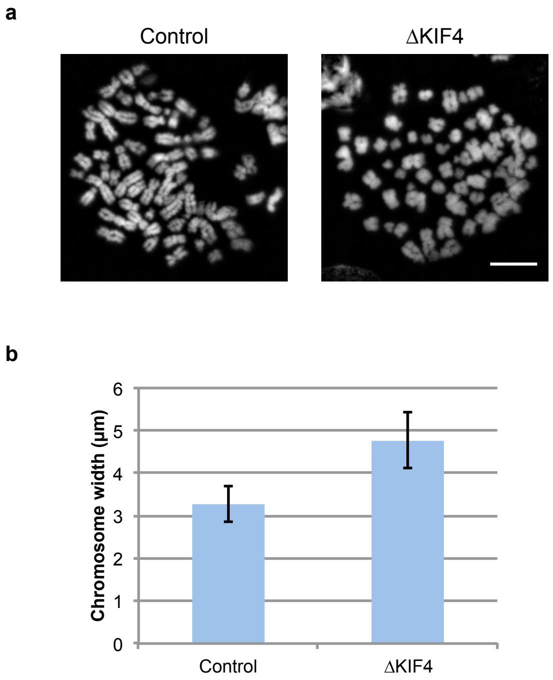

Supplementary Figure 5. Chromosome appearances from control and KIF4 depleted cells. a, Chromosome spreads were prepared from control and KIF4 depleted HeLa-wt cells. Scale bar, 10 μm. DNA was counterstained with Hoechst 33342. **b**, Measurement of chromosome width of mock and KIF4 depleted metaphase chromosomes. N = 31 and 51, respectively. Bar denotes the mean; error bar denotes standard deviation.

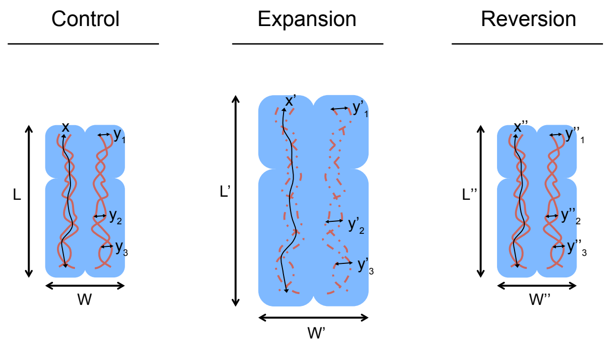

After expansion:
Reletive chromosome length = L'/L
Reletive chromosome width = W'/W
Reletive scaffold length = x'/x
Reletive scaffold width = $\Sigma y'_n/\Sigma y_n$

After reversion:
Reletive chromosome length = L''/L
Reletive chromosome width = W''/W
Reletive scaffold length = x''/x
Reletive scaffold width = $\Sigma y''_n/\Sigma y_n$

Supplementary Figure 6. Measurement of chromosome and chromosome scaffold length and width during expansion and reversion condition. To obtain chromosome or scaffold length and width of the chromosome during control, expansion and reversion states, we measured chromosome or scaffold dimensions at each state as shown in this figure. L, length of chromosome; W, width of chromosome; x, length of scaffold; y, width of scaffold. To obtain relative chromosome or scaffold length and width, we calculate chromosome or scaffold length and width using formula as presented in this figure.

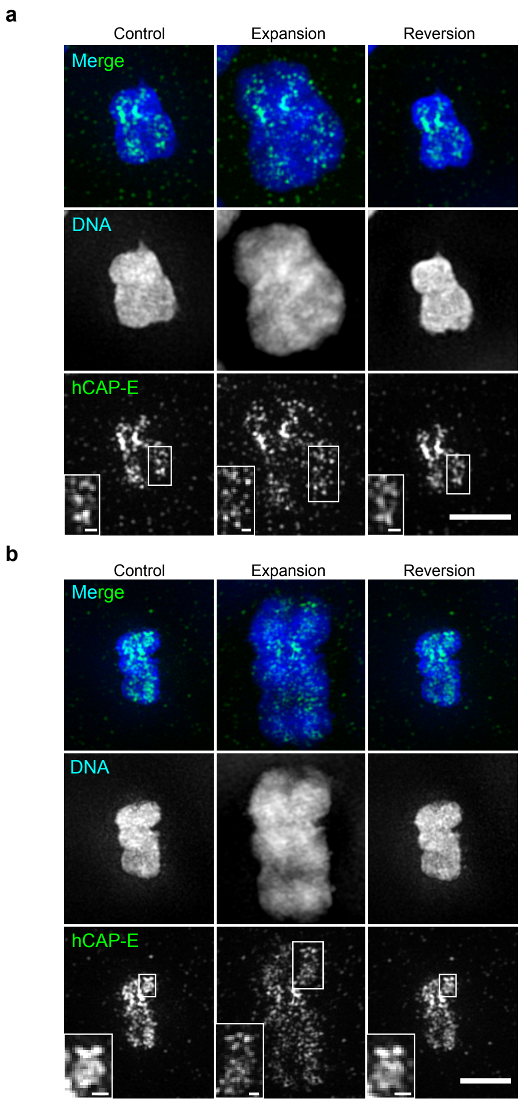

Supplementary Figure 7. Chromosome and chromosome scaffold alterations during chromosome expansion with 1 mM EDTA and reversion by univalent cations. Unfixed PA chromosomes were immunolabelled with hCAP-E and similar assay as Fig. 4a was performed using H-K and H-Na as reversible buffers. **a-b**, Maximum intensity projections of z-stack images obtained by 3D-SIM of the unfixed PA chromosome. Scale bars, 2 µm. Magnified views of white boxes are shown in insets. Scale bars, 250 nm. Chromosome and chromosome scaffold exhibit increasing length and width after subjection to 1 mM EDTA solution and capably restored to original morphology by subjection to either H-K or H-Na, suggesting that the distribution of scaffold proteins was not dependent on the kind of cation.
